# Supplementary figures and images for: Structure of the human heparan-α-glucosaminide N-acetyltransferase (HGSNAT)
Source: eLife. 2024 Aug 28;13:RP93510. doi: 10.7554/eLife.93510 (PMC11357348; doi:10.7554/eLife.93510)

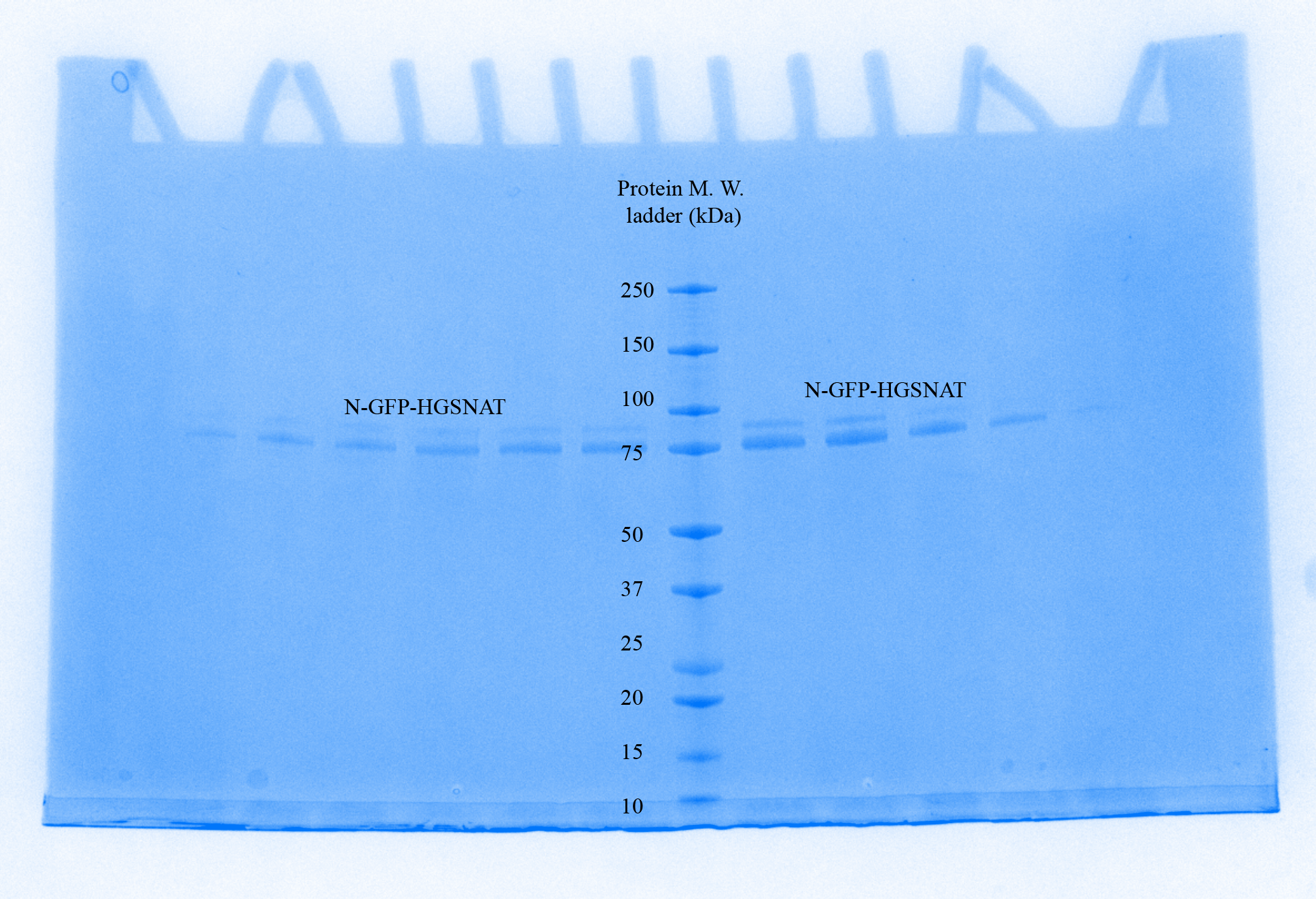

Supplement: Figure 1—figure supplement 1—source data 1. [file elife-93510-fig1-figsupp1-data1.zip › Figure1-Figure_supplement_1M-Source_Data_1_labeled.tif]

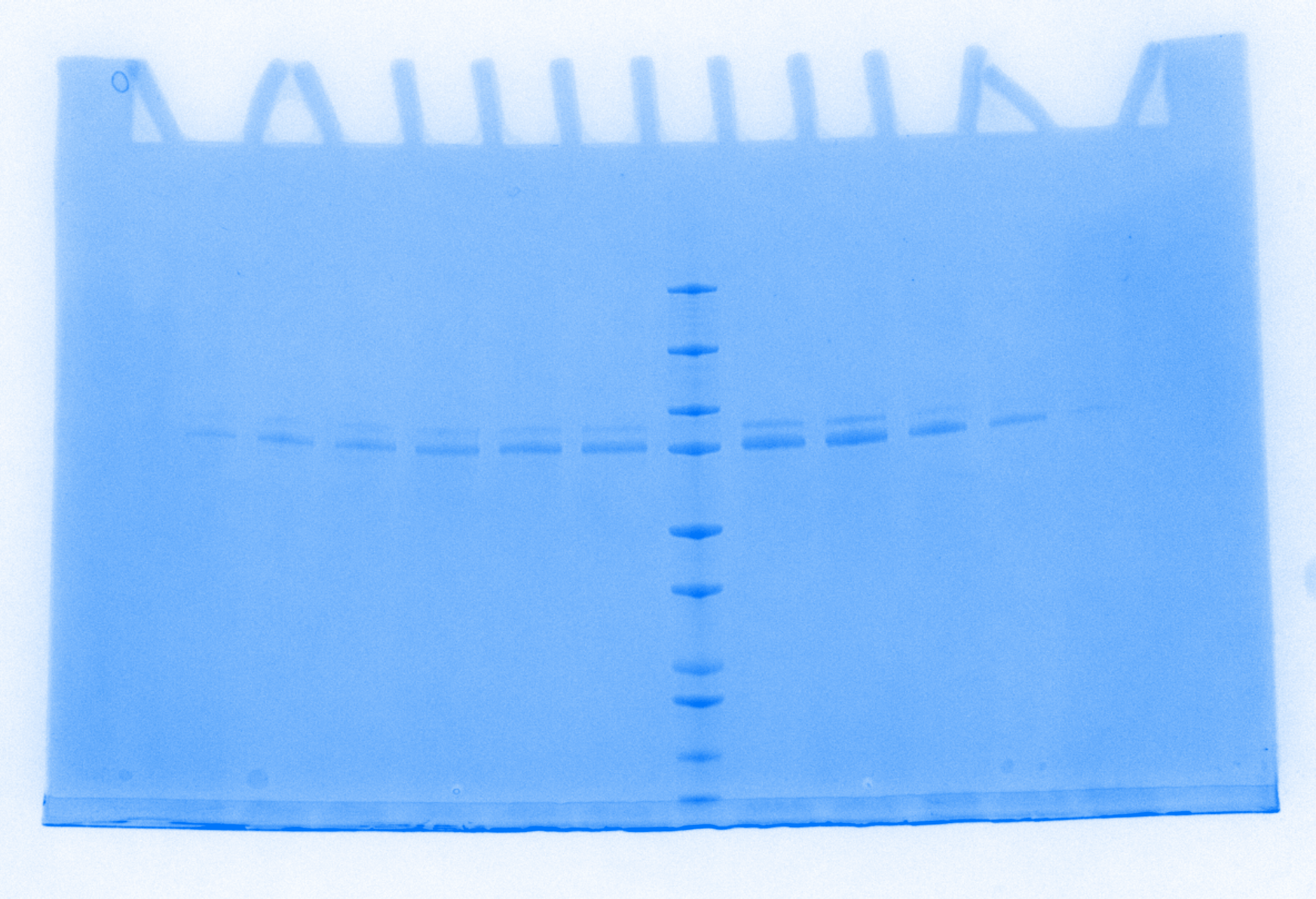

Supplement: Figure 1—figure supplement 1—source data 2. [file elife-93510-fig1-figsupp1-data2.zip › Figure1-Figure_supplement_1M-Source_Data_2.tif]
