# Supplementary material for: Structure of the human heparan-α-glucosaminide N-acetyltransferase (HGSNAT)
Source: eLife. 2024 Aug 28;13:RP93510. doi: 10.7554/eLife.93510 (PMC11357348; doi:10.7554/eLife.93510)
Supplement: Supplementary file 1. — The poor % identity (<20%) and low % sequence alignment suggests that there are no available structures of homologs of HGSNAT. Low mean RMSD of hits obtained using LD of HGSNAT as input suggests that LD is like some of the existing β-sandwiches, but TMD of HGSNAT is a novel fold. [file elife-93510-supp1.docx]

**Supplementary file 1**

**Homologs of TMD and LD of HGSNAT found in a Dali search**

| **PDB ID** | **RMSD (Å)** | **Number of equivalent residues** | **Total number of residues** | **% Identity** | **Protein** |
| --- | --- | --- | --- | --- | --- |
| *Homologs of TMD* | | | | | |
| 5LNK | 5.8 | 84 | 175 | 17 | Mitochondrial complex 1, 51 kDa subunit |
| 5CE3 | 4.3 | 93 | 598 | 16 | Actin |
| 6B2Z | 9.1 | 87 | 249 | 15 | Mitochondrial ATP synthase subunit C |
| 6HV6 | 4 | 66 | 293 | 15 | PatoxP toxin |
| 6KKK | 5.9 | 133 | 380 | 14 | Sugar efflux transporter SotB |
| *Homologs of LD* | | | | | |
| 6CZT | 2 | 81 | 82 | 15 | AlgF, alginate biosynthesis protein |
| 6IXH | 3.1 | 87 | 123 | 14 | Type VI secretion system core complex TSSJ |
| 6G7G | 3.1 | 88 | 115 | 13 | S-protein homolog (SPH) 15 |
| 5A9I | 2.3 | 95 | 194 | 12 | ECD of PepT2 |
| 5AMT | 3.7 | 84 | 105 | 12 | Intracellular growth locus E (IglE) |

Dali (**D**istance M**a**trix A**li**gnment) web server was used to search the existing database of known structures to find homologs of HGSNAT (Holm *et al,* 2023). The poor % identity (<20%) and low % sequence alignment suggests that there are no available structures of homologs of HGSNAT. Low mean RMSD of hits obtained using LD of HGSNAT as input suggests that LD is like some of the existing β-sandwiches, but TMD of HGSNAT is a novel fold.
