# Supplementary material for: Structure of the human heparan-α-glucosaminide N-acetyltransferase (HGSNAT)
Source: eLife. 2024 Aug 28;13:RP93510. doi: 10.7554/eLife.93510 (PMC11357348; doi:10.7554/eLife.93510)
Supplement: Supplementary file 2. — FoldX web server was used to predict relative mutant stability (Schymkowitz et al., 2005). Positive total energy value indicates destabilization, with greater values meaning lower stability. Nonsense mutations indicated in the Figure 4 as black were not included in FoldX calculations. Polymorphisms are italicized. All other mutants listed are missense mutations (Canals et al., 2011; Fan et al., 2006; Fedele and Hopwood, 2010; Feldhammer et al., 2009a; Feldhammer et al., 2009b; Hrebícek et al., 2006; Huizing and Gahl, 2020). [file elife-93510-supp2.docx]

**Supplementary file 2**

**List of HGSNAT mutations implicated in MPS IIIC**

| **Mutation** | **Total energy (kcal/mol)** | **Region of the protein** |
| --- | --- | --- |
| G423W | 41.0 | LD-TMD interface |
| G424V | 18.8 |  |
| G424S | 11.8 |  |
| G262R | 7.5 |  |
| C76F | 5.0 |  |
| N273K | -0.6 |  |
| G133A | -1.4 |  |
| P283L | 3.0 | Catalytic core |
| R344H | 2.0 |  |
| R344C | 2.0 |  |
| E471K | 1.4 |  |
| N258I | -0.1 |  |
| G486E | 17.9 | Scaffold domain |
| M482K | 3.2 |  |
| S518F | 2.5 |  |
| W403C | 2.2 |  |
| A489E | 1.1 |  |
| ***K523Q*** | 0.9 |  |
| S539C | 0.7 |  |
| S541L | -0.2 |  |
| ***V481L*** | -0.3 |  |
| L445P | 5.9 | Other regions |
| L113P | 4.9 |  |
| L137P | 3.4 |  |
| A54V | 2.9 |  |
| ***A615T*** | 2.3 |  |
| Y627C | 1.4 |  |
| D562V | 1.2 |  |
| ***P237Q*** | 0.1 |  |
| P571L | -0.2 |  |
| G173D | -0.4 |  |
| G173E | -0.9 |  |

FoldX web server was used to predict relative mutant stability (Schymkowitz *et al,* 2005). Positive total energy value indicates destabilization, with greater values meaning lower stability. Nonsense mutations indicated in the figure 4 as black were not included in FoldX calculations. Polymorphisms are italicized. All other mutants listed are missense mutations (Canals *et al,* 2011; Fan *et al,* 2006; Fedele & Hopwood, 2010; Feldhammer *et al,* 2009a; Feldhammer *et al,* 2009b; Hrebicek *et al,* 2006; Huizing & Gahl, 2020).
